# Migration of a Nasopharyngeal Airway Into the Gastrointestinal Tract During Endoscopic Retrograde Cholangiopancreatography: A Rare Complication in Obstructive Jaundice Management

**DOI:** 10.1155/crgm/2476267

**Published:** 2026-06-30

**Authors:** Xu Yan, Zhiqiang Zhou, Ailin Luo, Xue Zhang, Yilin Zhao

**Affiliations:** ^1^ Department of Anesthesiology, Key Laboratory of Geriatric Anesthesia and Perioperative Brain Health, and Wuhan Clinical Research Center for Geriatric Anesthesia, Tongji Hospital, Tongji Medical College, Huazhong University of Science and Technology, Wuhan, Hubei, China, hust.edu.cn

## Abstract

**Background:**

Obstructive jaundice often requires endoscopic retrograde cholangiopancreatography (ERCP) for diagnosis and management. Airway management during sedation in ERCP poses unique challenges, though device‐related complications are rarely reported.

**Case Presentation:**

A 35‐year‐old male with obstructive jaundice due to distal common bile duct stenosis underwent ERCP under sedation with propofol and oxycodone. A 16Fr silicone nasopharyngeal airway (NPA) was inserted due to mild upper airway obstruction. During the prolonged procedure (185 min), the NPA migrated unnoticed into the stomach, where it was discovered upon endoscope withdrawal and successfully retrieved using rat‐tooth forceps.

**Intervention:**

The NPA was removed without mucosal injury, and a biliary metal stent was deployed uneventfully.

**Outcome:**

The patient recovered without gastrointestinal or pulmonary complications, with normalized bilirubin levels at 1‐month follow‐up.

**Conclusion:**

This first reported case of NPA migration into the gastrointestinal tract during ERCP highlights the need for improved fixation, monitoring, and awareness of airway devices during prolonged endoscopic procedures.

## 1. Introduction

Obstructive jaundice is a clinical condition that occurs when there is a buildup of bilirubin in the bloodstream due to a blockage in the bile ducts, resulting in a yellowing of the skin and eyes. This condition can arise from several causes, such as gallstones, tumors, or strictures in the bile duct [1, 2]. The frequency of obstructive jaundice highlights the necessity for prompt diagnosis and treatment, especially in situations where endoscopic retrograde cholangiopancreatography (ERCP) is needed. ERCP is an essential procedure for diagnosing and managing biliary obstructions [3, 4]. However, it is important to note that ERCP can lead to complications, which may stem from technical challenges during the procedure. One such complication can involve the unintended movement of airway management devices, such as nasopharyngeal airways (NPAs).

In this report, we describe a distinctive case involving a 35‐year‐old male patient who developed obstructive jaundice and encountered an unusual complication during ERCP, specifically the migration of a NPA into the gastrointestinal tract. The patient presented with worsening jaundice, which laboratory tests confirmed by showing elevated levels of total bilirubin and alkaline phosphatase (ALP). Imaging studies further indicated stenosis in the distal common bile duct. The complexity of the ERCP procedure, which involved intricate guidewire manipulation and an extended duration, contributed to the oversight of the NPA’s migration. This situation highlights the significant risks associated with gastrointestinal complications that can arise from such procedural errors [5].

The clinical significance of this case is its novelty, as it marks the first reported instance of NPA migration into the stomach during an ERCP procedure. This incident underscores the potential risks linked to airway management devices used during sedation, highlighting the necessity for increased vigilance and enhanced intraoperative monitoring practices to prevent such events [5]. Additionally, gaining insight into the factors that lead to device migration can guide the development of future procedural protocols, ultimately improving patient safety and outcomes.

As gastroenterology advances, it is crucial to understand the complexities involved in managing obstructive jaundice and the potential risks associated with various procedures. This case highlights the significance of identifying uncommon complications and emphasizes the need for continuous research and improvement of endoscopic techniques. Such efforts are essential to uphold the highest standards of patient care and safety.

## 2. Case Presentation

### 2.1. Patient Information

This report describes a 35‐year‐old male patient who presented with progressive jaundice lasting one week. Laboratory examinations revealed total bilirubin levels at 186 μmol/L, direct bilirubin at 145 μmol/L, ALP at 452 U/L, and *γ*‐glutamyl transferase (GGT) at 380 U/L. Abdominal MRI/MRCP indicated distal common bile duct stenosis accompanied by intra‐ and extrahepatic duct dilation. The patient was diagnosed with obstructive jaundice of unknown etiology and was scheduled for an ERCP to perform a biopsy and place a covered metal stent. The preoperative assessment categorized the patient as ASA II, with a body mass index (BMI) of 22.3 kg/m^2^, modified Mallampati Class I, and normal cardiopulmonary and coagulation parameters, indicating no anticipated difficulty in airway management.

### 2.2. Clinical Findings

**FIGURE 1:**
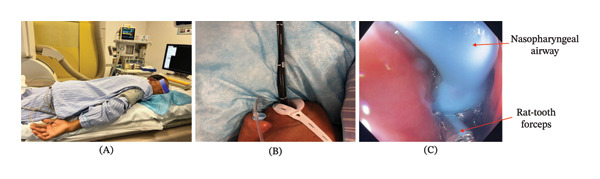
Patient positioning during endoscopic retrograde cholangiopancreatography (ERCP). (A) The patient is placed in the standard right semi‐prone position with the left chest elevated 30 degrees. (B) Use of a nasopharyngeal airway alleviates upper airway obstruction. (C) The nasopharyngeal airway migrated into the stomach.

During the ERCP procedure, the patient was positioned in a standard right semiprone position with the left chest elevated 30° (Figure 1). Routine monitoring was implemented, including ECG showing a heart rate of 72 bpm, noninvasive blood pressure recording 128/76 mmHg, and oxygen saturation at 98%. The sedation was maintained through intravenous administration of oxycodone (4 mg, 0.05 mg/kg) and a target‐controlled infusion of propofol (effect‐site concentration of 2.5 μg/mL), allowing the patient to maintain spontaneous respiration with a respiratory rate of 14–18 breaths per minute. During the ERCP procedure, the patient was placed in the standard right semiprone position with the left chest elevated 30°. Following the identification of mild upper airway obstruction (snoring and paradoxical breathing), a 16Fr silicone NPA was inserted unilaterally into the right nostril. The NPA was not sutured or taped to the skin, and water‐soluble lubricant was applied for insertion. No additional fixation device was used.

### 2.3. Diagnostic Assessment

The ERCP procedure advanced to the duodenal descending segment; however, repeated slippage of the guidewire hindered biliary cannulation, leading to conversion to endoscopic ultrasound‐guided biliary drainage (EUS‐BD). During the procedure, intraoperative CO_2_ pneumoperitoneum developed, causing abdominal distension that was relieved via 18G needle decompression. The prolonged endoscopic maneuvers, including repeated adjustments and guidewire manipulations, extended the total procedure duration to 185 min. The migration of the NPA into the stomach during this period is attributed to a combination of factors: the prolonged procedure time; the semiprone position, which may have altered pharyngeal anatomy and reduced natural resistance to NPA dislodgment; mechanical forces from endoscope and guidewire movements transmitted through the pharynx, potentially pushing the NPA downward; excessive lubrication combined with the smooth silicone surface of the NPA, reducing friction with the nasal and pharyngeal mucosa; and a lack of periodic verification of NPA position, as attention was focused on the challenges of biliary cannulation. Upon withdrawal of the endoscope, a light‐blue tubular foreign body measuring 15 cm was located along the greater curvature of the gastric body, later confirmed to be the dislodged NPA tip, identifiable by its manufacturer markings.

### 2.4. Therapeutic Intervention

The NPA was successfully retrieved using rat‐tooth forceps. Following this, an 8‐mm × 8 cm covered biliary metal stent was deployed without complication. Throughout the procedure, the patient’s vital signs remained stable, with a heart rate of 68 bpm, blood pressure of 115/70 mmHg, and oxygen saturation at 99%.

### 2.5. Follow‐Up and Outcomes

Postoperatively, the patient was moved to the general ward for continuous monitoring, which included 24‐h averages of heart rate at 66 ± 4 bpm, blood pressure at 122/68 ± 6/4 mmHg, and oxygen saturation between 97% and 99%. Hourly assessments of the abdomen indicated normal bowel sounds (3‐4 per minute) without tenderness or rigidity. Gastrointestinal integrity was confirmed with hemoglobin levels between 132 and 135 g/L and 3‐day fecal occult blood tests ruling out mucosal injury. A postoperative chest X‐ray taken six hours later excluded the presence of aspiration pneumonia, with a respiratory rate between 12 and 16 per minute. On Postoperative Day 3, total bilirubin levels decreased to 89 μmol/L, and nasobiliary cholangiography confirmed appropriate stent placement without contrast leakage. The patient resumed oral intake within 12 h postsurgery and transitioned to a regular diet by 48 h, experiencing no throat pain, nausea, or vomiting. Discharge occurred on Day 5, and a 1‐month follow‐up revealed resolved jaundice, with total bilirubin levels at 18 μmol/L and no delayed complications.

This case highlights the rare but significant complication of NPA migration during ERCP, emphasizing the necessity for increased intraoperative vigilance, proper device selection, and preventive measures to enhance patient safety. Further research is needed to refine management protocols and mitigate the occurrence of such complications.

## 3. Discussion

This case represents the first documented instance of NPA migration into the gastrointestinal tract during ERCP. While previous reports have described airway device displacement in other settings, such as during head and neck surgery or sleep endoscopy, the unique combination of ERCP positioning, prolonged procedure time, and mechanical interference from endoscopic instruments has not been previously highlighted [6–8].

### 3.1. Mechanisms of Migration

The semiprone position commonly used in ERCP alters the anatomical relationship between the nasopharynx and esophagus, potentially facilitating caudal displacement of an NPA [9]. Unlike supine positioning, where gravity and soft tissue compression may stabilize the device, the semiprone position reduces posterior pharyngeal wall contact, allowing easier distal migration. Additionally, repeated endoscope advancement and withdrawal generate oscillatory forces along the pharynx, which can progressively “walk” a lubricated, low‐friction NPA downward. This mechanical interference is rarely discussed in airway management literature but is highly relevant to ERCP, where endoscope manipulation is both vigorous and prolonged.

### 3.2. Comparison With Existing Literature

Previous studies on NPA complications have focused on insertion trauma, epistaxis, or incorrect sizing [10–12]. However, intraprocedural migration into the gastrointestinal tract has not been reported, likely due to underrecognition or underreporting. Our case suggests that ERCP poses specific risks that differ from other endoscopic procedures, such as upper endoscopy without biliary intervention, where procedure duration is shorter and patient positioning is typically left lateral.

### 3.3. Preventive Strategies

Based on this case, we recommend the following preventive measures: 1) Fixation of the NPA to the nasal ala or cheek using tape or a commercial device, especially when prolonged ERCP is anticipated. 2) Periodic visual or tactile verification of NPA position every 30–60 min during the procedure. 3) Use of shorter, flanged, or anatomically designed NPAs that resist distal displacement. 4) Avoidance of excessive lubrication; a minimal amount should be used solely for insertion. 5) Consideration of alternative airway devices, such as a supraglottic airway with gastric access, for high‐risk or long‐duration ERCP cases.

### 3.4. Limitations and Future Directions

This case is limited by its single‐event nature and the inability to directly observe the exact moment of migration. Future studies should consider prospective observational designs to assess NPA stability in different positions and during various endoscopic maneuvers. Development of a standardized protocol for airway device monitoring during ERCP is urgently needed.

## 4. Conclusion

This report highlights a rare but significant complication of NPA migration into the gastrointestinal tract during ERCP, a finding not previously documented. The mechanism likely involves a combination of semiprone positioning, prolonged procedure time, mechanical forces from endoscope manipulation, and inadequate device fixation. Increased awareness, routine position checks, and improved fixation strategies are essential to prevent such events and enhance patient safety.

## Author Contributions

Xu Yan and Zhiqiang Zhou were responsible for data acquisition. Xue Zhang drafted the article, while Ailin Luo and Yilin Zhao provided revisions to the manuscript. All authors participated in drafting and reviewing.

## Funding

This work was supported by a grant from the National Natural Science Foundation of China (No. 81500982), Chronic Disease Management Research Project of National Health Commission Capacity Building and Continuing Education Center (GWJJMB202510042082, GWJJMB202510042083), and Chinese Society of Anesthesiology Youth Anesthesiologist Research Fund (No. Z‐2017–24–2421), Beijing Health Alliance Charitable Foundation (No. S218), The 2025 Undergraduate Teaching Research Fund Project of the Second Clinical College, Tongji Hospital Affiliated with Tongji Medical College, Huazhong University of Science and Technology(TJSZ2025044).

## Disclosure

All authors approved the final version of the manuscript.

## Ethics Statement

This case report was approved by the Tongji Hospital Ethics Committee. All procedures were performed in accordance with the principles of the Declaration of Helsinki.

## Consent

Written informed consent was obtained from the patient for the publication of this case report and any accompanying images.

## Conflicts of Interest

The authors declare no conflicts of interest.

## Data Availability

All data generated or analyzed during this case report are included in this published article.
